# Unitarity and Page Curve for Evaporation of 2D AdS Black Holes

**DOI:** 10.3390/e24010101

**Published:** 2022-01-08

**Authors:** Mariano Cadoni, Andrea P. Sanna

**Affiliations:** 1 Dipartimento di Fisica, Università di Cagliari, Cittadella Universitaria, 09042 Monserrato, Italy; asanna@dsf.unica.it; 2 INFN, Sezione di Cagliari, Cittadella Universitaria, 09042 Monserrato, Italy

**Keywords:** black hole evaporation, Page curve, two-dimensional gravity models

## Abstract

We explore the Hawking evaporation of two-dimensional anti-de Sitter (AdS${}_{2}$), dilatonic black hole coupled with conformal matter, and derive the Page curve for the entanglement entropy of radiation. We first work in a semiclassical approximation with backreaction. We show that the end-point of the evaporation process is AdS${}_{2}$ with a vanishing dilaton, i.e., a regular, singularity-free, zero-entropy state. We explicitly compute the entanglement entropies of the black hole and the radiation as functions of the horizon radius, using the conformal field theory (CFT) dual to AdS${}_{2}$ gravity. We use a simplified toy model, in which evaporation is described by the forming and growing of a negative mass configuration in the positive-mass black hole interior. This is similar to the “islands” proposal, recently put forward to explain the Page curve for evaporating black holes. The resulting Page curve for AdS${}_{2}$ black holes is in agreement with unitary evolution. The entanglement entropy of the radiation initially grows, closely following a thermal behavior, reaches a maximum at half-way of the evaporation process, and then goes down to zero, following the Bekenstein–Hawking entropy of the black hole. Consistency of our simplified model requires a non-trivial identification of the central charge of the CFT describing AdS${}_{2}$ gravity with the number of species of fields describing Hawking radiation.

## 1. Introduction

Since the discovery of Hawking radiation [1,2], the information paradox for an evaporating black hole has been one of the most intriguing puzzles of fundamental theoretical physics. At the semiclassical level, unitarity of quantum mechanics seems to be lost when a black hole is formed from a collapsing pure quantum state and then completely evaporates, leaving behind only thermal Hawking radiation [3], described by a mixed quantum state (see, e.g., refs. [4,5,6] for reviews).

Over the years, several possibilities of addressing the problem have been put forward. Either information may be lost forever [7,8]—as firstly advocated by Hawking [3], whose argument, however, conflicted with energy conservation [9]—or one could have remnants [10] at the end of the evaporation, fuzzy structures at the horizon (fuzzball) [11] or, finally, information leaks out and is somehow encoded in the Hawking radiation.

In more recent times, the holographic principle [12], its explicit realization through the AdS/CFT correspondence [13,14,15,16] and the discussions triggered by the firewall argument [17,18] supported a solution of the information puzzle, which preserves the unitarity of quantum mechanics. In fact, the AdS/CFT correspondence implies that any gravitational bulk process in *D*-dimensions, such as black hole evaporation, can be holographically described in terms of a (D−1)-dimensional conformal field theory on the boundary, for which the evolution of quantum states is unitary. This suggests that the relevant gravitational degrees of freedom may be encoded on the boundary of a volume of the space-time rather than inside its bulk. This, in turn, allows for a microscopic explanation of the Bekenstein–Hawking black hole entropy (see, e.g., [16,19,20,21]), the backbone on which the “Central Dogma” (for an external observer, black hole quantum dynamics can be described as the unitary time evolution of $N∼S_{BH}$ quantum states, where $S_{BH}$ is the Bekenstein–Hawking black hole entropy [22]) of black hole information is based. 

It is worth mentioning that several alternative solutions to the paradox have been proposed, which do not explicitly rely on holography. Some of them introduce non-local effects in standard quantum field theory in a gravitational background [23,24,25,26]. Alternatively, they are based on more conservative approaches, such as the recovery of information through the reconstruction of the quantum correlations among Hawking particles (see, e.g., [27]), or on a Bohr-like quantum description of the black hole, inspired by the spectrum of quasi-normal modes (see [28] and references therein).

From a purely bulk perspective, one possible way to preserve unitarity of quantum mechanics during the evaporation is to assume that information leaks out from the black hole, encoded in Hawking radiation. Using quite general arguments of information theory, Don Page has shown this can only happen at late times, after the so-called Page time, when roughly half of the black hole has evaporated away. This process is characterized by a well defined pattern for the entanglement entropy (EE) of the radiation—the famous Page curve [29,30]. 

Very recent developments give further support to information conservation during the black hole evaporation process. They are based on a semiclassical approach to compute the entropy of Hawking radiation which consists in extremizing an entropy functional, expressed as the sum of the contribution of the entropy of bulk fields and that of several, disconnected regions, inside and outside the black hole, called “islands” [22,31,32]. The formula is manifestly a generalization of the holographic Ryu–Takayanagi formula [33,34] and is based on the idea of the “entanglement wedge reconstruction” [35,36,37,38], which allows computing the Page curve semiclassically by correctly keeping track of the entanglement structure of both the black hole and the radiation subsystems. At early times, before the Page time, the entanglement wedge of the black hole includes all the interior, with a neat separation between radiation and black hole degrees of freedom. The entropy of the radiation will, therefore, increase as Hawking quanta start leaking out. At late times, after the Page time, a contribution given by the islands, forming just behind the horizon, starts dominating. This determines the consequent decrease of the Page curve, signalizing the purification of the final state of the radiation. 

The new generalized entropy formula, however, does not tell where the information is encoded and how it manages to escape from the horizon. This is mainly due to the fact that the formula is built in terms of the low-energy gravitational theory and makes no reference to the underlying microscopic theory and to the would-be unitary dynamics of the $N∼S_{BH}$ quantum states building up the black hole. Another drawback of the generalized entropy formula is that computations are in general quite difficult to perform. 

In view of this state of the art, it is quite important to consider gravitational systems, for which we have at least an effective description of the underlying microscopic dynamics and in which the semiclassical black hole dynamics is simple enough to allow for an explicit analysis. 

The most natural candidates are the two-dimensional (2D) AdS (AdS${}_{2}$) black holes of Jackiw–Teitelboim (JT) dilaton gravity [39,40,41]. The latter represents one of the most studied 2D gravity models, as it allows describing, otherwise more difficult to capture, features of four-dimensional gravitational black holes [41,42] and has several features that make it suitable for the before-mentioned purposes. Owing to the fact that Hawking radiation has a purely topological origin in these models [43], the semiclassical dynamics of black hole solutions can be solved in closed analytical form. For small values of the 2D Newton constant $1/\phi_{0}$, they allow for an effective description in terms of a dual CFT with central charge $c=12\phi_{0}$. This enables a microscopic derivation of the BH entropy [21], so that the Central Dogma is based on solid ground. The black hole spectrum of the JT theory contains an energetically preferred, regular, ground state (the AdS${}_{2}$ space-time), a state with zero temperature and zero entropy [21,44], which is the perfect candidate for the end point of the evaporation process. Last but not least, the dual CFT description of JT black holes allows for an explicit computation of the black hole EE [45].

These nice features allow describing analytically the behavior of the entropy of both the black hole and the radiation and, consequently, to reconstruct the Page curve. In [37], for example, this is done by using the holographic entropy formula [33,46,47] and the entanglement wedge reconstruction idea, for a JT black hole coupled with a CFT${}_{2}$ matter sector with a higher-dimensional holographic dual. In particular, the latter allows connecting the interior of the black hole with the radiation subsystem, in the spirit of the “Einstein–Podolsky = Einstein–Podolsky–Rosen” (ER = EPR) conjecture [48,49,50]. The same technique is applied in [51] to derive the Page curve in the 2D Callan–Giddings–Harvey–Strominger (CGHS) model [52,53]. Finally, in [54], the Page curve for JT black holes is derived by considering the 2D gravitational model as the dimensional reduction of the 3D AdS gravity.

In this paper, we tackle the information problem for the JT black hole using an alternative approach. We investigate the semiclassical dynamics of JT black holes, coupled to conformal matter in the form of *N* massless scalar fields, including the backreaction of the geometry, and derive the Page curve for their EE in a closed form. We will do so without using either higher-dimensional theories or the generalized holographic entropy formula. We show that, working in the semiclassical approximation with backreaction, the end-point of the evaporation is the AdS${}_{2}$ space-time, endowed with a vanishing dilaton, i.e., a regular, singularity free, zero entropy space-time. This suggests unitary evolution and information conservation.

We proceed by computing the entanglement entropies of the black hole and the radiation as functions of the horizon radius. This is done using the effective description of AdS${}_{2}$ quantum gravity in terms of the dual CFT, i.e., in the large central charge regime, c≫1, of the CFT. The computation of the EE of the radiation is performed using a simplified toy model, in which black hole evaporation is described by the formation and growth of a negative mass configuration in the positive-mass black hole interior. This setup represents a rough, simplified version of the earlier-mentioned “islands” conjecture, which has been recently proposed to explain the Page curve for evaporating black holes (see, e.g., [22,31,32,51,54,55,56,57,58,59,60]). Unlike the semiclassical entropy formula used in the aforementioned papers, the EE formula used in this paper allows us to capture also contributions of purely quantum mechanical correlations between the interior and the exterior of the black hole, in line with the ER=EPR spirit as well. Moreover, these correlations arise in a natural and simple way in our model, without resorting to higher-dimensional duals.

In the final part of our paper, we compare the curve of the EE for Hawking radiation with those pertaining to the thermal entropy of the radiation and the Bekenstein–Hawking entropy of the black hole. The resulting Page curve for JT black holes is in agreement with unitary evolution. The entanglement entropy of the radiation initially grows, closely following a thermal behavior, reaches a maximum at the half-way point, and then goes down to zero, closely following the Bekenstein–Hawking entropy of the black hole during the final stages of the evaporation process. Basic principles of thermodynamics, together with the existence of a dual CFT description, imply a non-trivial identification of the central charge of the CFT describing AdS${}_{2}$ gravity with the number *N* of massless fields describing Hawking radiation.

The structure of this paper is as follows. In Section 2, we briefly review the classical and semiclassical properties of JT black holes coupled with conformal matter, focusing on the conformal anomaly and backreaction effects of the geometry. We investigate the semiclassical dynamics of the model in Section 3, by considering the evaporation process, both in static coordinates and in terms of boundary dynamics. The calculation of the EE associated with the JT black hole in [45] is reviewed in Section 4. In Section 5, we discuss the information flow during the black hole evaporation and present the main results of this paper concerning the Page curve for the JT black hole. We compare the EE of the radiation with the thermal entropies of the radiation and of the black hole. We also derive the relationship between the central charge of the CFT dual to AdS${}_{2}$ gravity and the number of species of matter fields in the Hawking radiation. Finally, in Section 6, we state our conclusions.

## 2. 2D AdS Black Holes

In this paper, we consider 2D AdS black holes. The simplest gravity model allowing for this kind of solutions is JT gravity, described by the action

(1) $$\begin{array}{c} S_{\mathrm{JT}}=\frac{1}{2\pi }\int d^{2}x\sqrt{-g}\;\phi \left(R+2\Lambda^{2}\right)+S_{\mathrm{matter}}, \end{array}$$

where ϕ is a scalar field (the dilaton), playing the role of the inverse coordinate-dependent 2D Newton constant, *R* is the 2D Ricci scalar, $\Lambda^{2}$ the cosmological constant and $S_{\mathrm{matter}}$ is the action for matter fields. A slight generalization of this model has been proposed by Almheiri and Polchinski (AP) [61], by adding a constant term α to the dilaton potential:

(2) $$\begin{array}{c} V\left(\phi \right)=2\Lambda^{2}\left(\alpha -\phi \right). \end{array}$$

Thus, the JT model can be considered as a particular case (α=0) of the AP model. We will make use of this feature in the following section.

### 2.1. Classical Solutions in Absence of Matter

In absence of matter, JT gravity admits asymptotically AdS${}_{2}$ black holes as solutions [43]. In the Schwarzschild gauge, the metric and the dilaton read

(3) $$\begin{array}{c} ds^{2}=-\left(\Lambda^{2}r^{2}-a^{2}\right)dt^{2}+{\left(\Lambda^{2}r^{2}-a^{2}\right)}^{-1}dr^{2},\;\phi \left(r\right)=\phi_{0}\Lambda r, \end{array}$$

where $\phi_{0}$ and $a^{2}$ are integration constants related to the Arnowitt–Deser–Misner (ADM) mass of the solution:

(4) $$\begin{array}{c} M=\frac{1}{2}\phi_{0}a^{2}\Lambda =\frac{1}{2}\phi_{0}\Lambda^{3}r_{h}^{2}. \end{array}$$

$a^{2}>0$, $a^{2}=0$ and $a^{2}<0$ represent, respectively, a space-time with a positive ADM mass, with Killing horizon at $r=r_{h}=a/\Lambda$, the AdS vacuum (zero ADM mass) and a space-time with a negative ADM mass. Adopting the nomenclature of [43], we will refer to these as AdS${}_{+}$ ($a^{2}>0$), AdS${}_{0}$ ($a^{2}=0$) and AdS${}_{-}$ ($a^{2}<0$). These three solutions represent different parameterizations, covering different regions of the same manifold, as they are connected with each other by coordinate transformations. This means that the *local* properties of the space-time described by the metric (3) are the same, independently from the value of $a^{2}$. Therefore, the three space-times can be maximally extended to obtain full AdS${}_{2}$, which has no horizon and is geodesically complete. Nevertheless, AdS${}_{+}$ can be interpreted as a 2D black hole with an event horizon at $r=r_{h}$, if one takes into account the physical meaning of the dilaton as the (coordinate-dependent) inverse 2D Newton constant, demanding ϕ⩾0. This requirement implies the existence of a space-time singularity at r=0, where the 2D Newton constant diverges. The ϕ=0 line has to be considered as an inner boundary of the space-time, whose existence allows one to consider AdS${}_{+}$ as a black hole, AdS${}_{-}$ as a space-time containing naked singularities and AdS${}_{0}$ as the ground state, zero mass solution [43] (a similar conclusion can be reached if one considers the JT model as originated from spherical dimensional reduction of 3D Bañados–Teitelboim–Zanelli (BTZ) [62] or higher dimensional [43] black holes. In this case, the positivity condition ϕ⩾0 is required by the identification of ϕ with the radius of the compactified sphere).

Once we interpret AdS${}_{+}$ as a black hole space-time, it is natural to associate thermodynamic properties to it, i.e., a temperature $T_{H}$ and an entropy $S_{BH}$:

(5) $$T_{H}=\frac{a\Lambda }{2\pi }=\frac{r_{h}\Lambda^{2}}{2\pi }=\frac{1}{2\pi }\sqrt{\frac{2M\Lambda }{\phi_{0}}};$$

(6) $$S_{BH}=2\pi \Lambda \phi_{0}r_{h}=4\pi \sqrt{\frac{\phi_{0}M}{2\Lambda }}=2\pi \phi \left(r_{h}\right).$$

The AdS${}_{0}$ vacuum solution with a linear dilaton, given by Equation (3) with a=0, termed linear dilaton vacuum (LDV) in [44], is not the only M=0 vacuum of the model. The theory allows also for full AdS${}_{2}$ space-time solution endowed with a constant, identically vanishing, dilaton,

(7) $$\begin{array}{c} \phi =0, \end{array}$$

which has been called the constant dilaton vacuum (CDV) solution in [44] (similarly, the AP model admits both AdS black hole solutions with a linear varying and a constant non-vanishing dilaton [61]).

At first sight, the CDV and the LDV seem degenerate in energy (they both have zero ADM mass), but closer inspection reveals that they are separated by a mass gap $M_{\mathrm{gap}}=\Lambda /\left(2\pi^{2}\phi_{0}\right)$[44,61]. Indeed the CDV, being full AdS${}_{2}$ space-time, does not admit finite energy excitation [63]. The issue can be better understood if we consider the JT model as a particular case of the AP model, where the two vacua are connected by an interpolating solution $\phi =\alpha^{2}+\phi_{0}\Lambda r$. Following [44], we can now remove the apparent degeneracy between the CDV and the LDV and study the thermodynamic behavior of the two vacua by looking at the free energy difference $\Delta F=F^{\mathrm{LDV}}-F^{\mathrm{CDV}}$. At $T_{H}\neq 0$, $\Delta F=-\frac{2\pi^{2}\phi_{0}}{\Lambda }T_{H}^{2}<0$ [44], which tells us that the LDV is thermodynamically favored. However, at T=0, for the CDV ($\phi_{0}\to 0$), $M_{\mathrm{gap}}$ diverges and one has $\Delta F=\frac{\alpha^{4}\Lambda }{2\phi_{0}}\to \infty$ [44]. The LDV is therefore not thermodynamically stable at zero temperature: a phase transition occurs, which drives the system down to the CDV.

Let us now describe the black hole solution in the conformal gauge, using light-cone coordinates $x^{\pm }$,

(8) $$\begin{array}{c} ds^{2}=-e^{2\rho \left(x^{+},x^{-}\right)}dx^{+}dx^{-},\;x^{\pm }=x^{0}\pm x^{1}, \end{array}$$

which will be also used to describe the coupling of our model to matter fields. The field equations and constraints, stemming from the action (1), in absence of matter, read now

(9) $$\partial_{+}\partial_{-}\rho =-\frac{\Lambda^{2}}{4}e^{2\rho };$$

(10) $$\partial_{+}\partial_{-}\phi =-\frac{\Lambda^{2}}{2}\phi e^{2\rho };$$

(11) $$\partial_{+}^{2}\phi ---2\partial_{+}\phi \partial_{+}\rho =0;$$

(12) $$\partial_{-}^{2}\phi ---2\partial_{-}\phi \partial_{-}\rho =0.$$

They are solved by

(13) $$e^{2\rho }=\frac{4}{\Lambda^{2}}{\left(x^{-}-x^{+}\right)}^{-2};$$

(14) $$\phi =\frac{b+c\left(x^{+}+x^{-}\right)+dx^{+}x^{-}}{x^{-}-x^{+}},$$

where a,b,c are constants. The metric part of the solution (13) has an SL(2,R) isometry, i.e., it is invariant under the transformations:

(15) $$x^{\pm }\to \frac{\alpha x^{\pm }+\beta }{\gamma x^{\pm }+\delta },\;\mathrm{with}\;\alpha \delta -\beta \gamma =1.$$

By exploiting this invariance, the dilaton (14) can be recast as [43]:

(16) $$\begin{array}{c} \phi =\frac{2\phi_{0}}{\Lambda }\frac{1-\frac{a^{2}\Lambda^{2}}{4}x^{+}x^{-}}{x^{-}-x^{+}}. \end{array}$$

The coordinate transformations relating the solutions written in the Schwarzschild gauge (3) to those written in the the conformal gauge (13) and (14) are:

(17) $$\left\{\begin{array}{cc} \; & x^{+}=\frac{2}{a\Lambda }\tanh\left\{\frac{a\Lambda }{2}t-\frac{1}{2}\mathrm{arcsinh}\left[{\left(\frac{\Lambda^{2}r^{2}}{a^{2}}-1\right)}^{-1/2}\right]\right\},\\ \; & x^{-}=\frac{2}{a\Lambda }\tanh\left\{\frac{a\Lambda }{2}t+\frac{1}{2}\mathrm{arcsinh}\left[{\left(\frac{\Lambda^{2}r^{2}}{a^{2}}-1\right)}^{-1/2}\right]\right\}. \end{array}\right.$$

The AdS${}_{0}$ solution is easily obtained by taking the zero mass limit, a→0, of Equation (16)

(18) $$\begin{array}{c} \phi =\frac{2\phi_{0}}{\Lambda }{\left(x^{-}-x^{+}\right)}^{-1}, \end{array}$$

while the metric conformal factor (13) remains unchanged. This is the consequence of a general and peculiar feature of the JT theory, which is easily seen from Equation (9): the classical dynamics of the conformal factor is independent from the dilaton. This implies that the dynamics of the model is fully encoded in the scalar field, which determines the global properties of the space-time through the evolution of the ϕ=0 space-time singularity. As we will show in the next section, this feature remains true also when we couple the gravity model to conformal matter both at the classical and semiclassical level.

**Figure 1:**
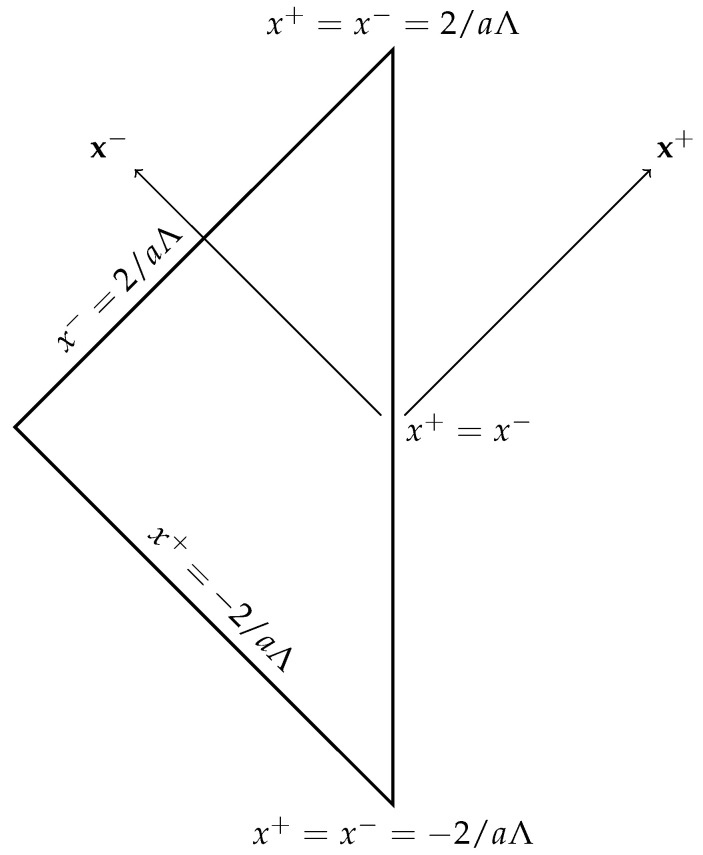
Penrose diagram of 2D AdS${}_{+}$. The future and past asymptotic singularities are highlighted, corresponding to the two vertices on the line $x^{+}=x^{-}$ of the diagram. The two tilted lines correspond to the future $x_{H}^{-}=2/a\Lambda$ and past $x_{H}^{+}=-2/a\Lambda$ event horizons.

Let us briefly discuss the Penrose diagram of our space-time. In light-cone coordinates, full AdS${}_{2}$ space-time has two disconnected parts, which are represented by two wedges in the Penrose diagram. The asymptotic conformal boundary $x^{+}=x^{-}$ of the space-time is timelike. AdS${}_{+}$ is half of AdS${}_{2}$, which in this paper is taken as the left wedge, following the convention of [43] (see Figure 1). This means that we are considering the region $x^{-}⩾x^{+}$ of AdS${}_{2}$. The event horizon, $r=r_{h}=a/\Lambda$ in Schwarzschild coordinates, in light-cone coordinates corresponds to:

(19) $$x_{H}^{+}=-\frac{2}{a\Lambda };\;x_{H}^{-}=\frac{2}{a\Lambda }.$$

Considering the solution (16), the space-time singularity occurs at $1-\frac{a^{2}\Lambda^{2}}{4}x^{+}x^{-}=0$. The singularity is always shielded by the event horizons (19). The black hole interior corresponds to the region $x^{-}⩾2/a\Lambda$ and $x^{+}⩽-2/a\Lambda$. If one uses light-cone coordinates, so that the scalar ϕ takes the form (14), the singularity is visible for an asymptotic timelike observer sitting on the conformal asymptotic space-time boundary. He/she will hit the singularity at finite time, $x^{-}=-2/a\Lambda$ (past singularity) and $x^{-}=2/a\Lambda$ (future singularity), at least for a finite non-vanishing value of *M*. However, ϕ is not form-invariant under the isometric SL(2, R) transformation (15) and the presence of the future timelike singularity in the asymptotic boundary can be avoided by choosing an appropriate light-cone frame, which removes the $x^{+}x^{-}$ term in the dilaton solution (14). In fact, using an appropriate SL(2, R) transformation, the dilaton solution (14) may be written as follows,

(20) $$\phi =\frac{2\phi_{0}}{\Lambda }\frac{1+\frac{a\Lambda }{2}\left(x^{-}+x^{+}\right)}{x^{-}-x^{+}}.$$

In this new light-cone frame, the singularity trajectory ϕ=0 is

(21) $$x^{-}=-\frac{2}{a\Lambda }-x^{+},$$

which gives only a past singularity $x^{-}=-\frac{1}{a\Lambda }$, once evaluated on the timelike asymptotic boundary $x^{-}=x^{+}$. In Section 3.2, we will show that the SL(2, R) isometry of the metric can also be used to remove the asymptotic, future timelike singularity in presence of Hawking radiation and related backreaction of the geometry.

### 2.2. Coupling to Matter, Conformal Anomaly and Evaporation

Let us now couple our gravity model to matter fields, quantize the latter in the semiclassical approximation and include the backreaction of the geometry.

We consider the matter sector in the form of *N* massless scalar fields $f_{i}$, minimally coupled to gravity, described by the classical, conformally invariant action:

(22) $$\begin{array}{c} S_{\mathrm{matter}}=-\frac{1}{4\pi }\int d^{2}x\sqrt{g}\;\sum_{i=1}^{N}{\left(\nabla f_{i}\right)}^{2}. \end{array}$$

Quantization of the matter fields and backreaction of the geometry is studied at the semiclassical level by considering the quantization of the CFT matter on the curved, classical 2D gravitational background. This implies a non-zero trace of the (classically traceless) stress-energy tensor for the matter fields (conformal anomaly) [64]. For *N* massless fields $f_{i}$ in two space-time dimensions, the conformal anomaly reads $\langle T_{\mu }^{\mu }\rangle =\frac{N}{12}R$, which can be accounted for by adding a non-local Polyakov–Liouville term in the JT action (1)

(23) $$\begin{array}{c} S_{\mathrm{anomaly}}=-\frac{N}{96\pi }\int d^{2}x\sqrt{g}\;R{□}^{-1}R, \end{array}$$

where ${□}^{-1}$ is the inverse of the Laplacian. The field equations and the constraints, in light-cone coordinates become local and are given by

(24) $$\partial_{+}\partial_{-}\rho =-\frac{\Lambda^{2}}{4}e^{2\rho };$$

(25) $$\partial_{+}\partial_{-}\phi =-\frac{\Lambda^{2}}{2}\left(\phi -\frac{N}{24}\right)e^{2\rho };$$

(26) $$\partial_{+}^{2}\phi ---2\partial_{+}\phi \partial_{+}\rho =-\frac{1}{2}\sum_{i=1}^{N}\partial_{+}f_{i}\partial_{+}f_{i}+\frac{N}{12}\left[{\left(\partial_{+}\rho \right)}^{2}-\partial_{+}^{2}\rho +t_{+}\left(x^{+}\right)\right];$$

(27) $$\partial_{-}^{2}\phi ---2\partial_{-}\phi \partial_{-}\rho =-\frac{1}{2}\sum_{i=1}^{N}\partial_{-}f_{i}\partial_{-}f_{i}+\frac{N}{12}\left[{\left(\partial_{-}\rho \right)}^{2}-\partial_{-}^{2}\rho +t_{-}\left(x^{-}\right)\right];$$

(28) $$\partial_{+}\partial_{-}f_{i}=0,$$

where $t_{\pm }\left(x^{\pm }\right)$ are integration functions, which have to be determined by imposing appropriate boundary conditions.

As anticipated in the previous section, we see that, also in the semiclassical treatment, the conformal factor of the metric ρ is insensitive to the presence of matter, the dilaton and backreaction effects. All dynamical information on the evolution of the semiclassical system is completely encoded in the solution for the dilaton, which determines the evolution of the space-time boundary at ϕ=0.

Another striking feature of Equations (24)–(28) is the fact that, at the level of the field equations, the conformal anomaly, i.e., semiclassical quantum effects, can be reabsorbed by means of a translation of the dilaton. In fact, by performing the translation

(29) $$\phi =\varphi +\frac{N}{24},$$

and using the solution of Equation (24), Equations (24)–(28) reduce to

(30) $$\partial_{+}\partial_{-}\rho =-\frac{\Lambda^{2}}{4}e^{2\rho };$$

(31) $$\partial_{+}\partial_{-}\varphi =-\frac{\Lambda^{2}}{2}\varphi e^{2\rho };$$

(32) $$\partial_{+}^{2}\varphi ---2\partial_{+}\varphi \partial_{+}\rho =-\frac{1}{2}\sum_{i=1}^{N}\partial_{+}f_{i}\partial_{+}f_{i}+\frac{N}{12}t_{+};$$

(33) $$\partial_{-}^{2}\varphi ---2\partial_{-}\varphi \partial_{-}\rho =-\frac{1}{2}\sum_{i=1}^{N}\partial_{-}f_{i}\partial_{-}f_{i}+\frac{N}{12}t_{-};$$

(34) $$\partial_{+}\partial_{-}f_{i}=0,$$

which coincides with the classical one (10), apart from the dilaton translation and the presence of the functions $t_{\pm }$. The effect of the conformal anomaly is just a translation of the space-time boundary, which now is located at ϕ=N/24 and the appearance of the functions $t_{\pm }$ in the field equations.

These functions $t_{\pm }$ play an important role. Their presence is a consequence of the anomalous transformation law of $T_{\pm \pm }$, which is given in terms of the Schwarzian derivative of light-cone transformation function (see Equation (17)). Usually, the $t_{\pm }$ are fixed by imposing boundary conditions on Hawking radiation at past infinity (see, e.g., [52]).

Let us conclude by noticing that the backreaction effects can be reabsorbed by the translation (29) also at the level of the action. This can be done by including a purely topological term $\Phi_{0}R$ into the action (1), with $\Phi_{0}$ constant, and performing the shift $\Phi_{0}={\hat{\Phi }}_{0}+\frac{N}{24}$ together with the translation (29).

## 3. Black Hole Evaporation

In the previous section, we showed that the classical and semiclassical dynamics of our model is independent from the metric, but is fully encoded in the solution for the dilaton. We have therefore only two options to describe the evaporation process: (1) we use a static coordinate patch covering the black hole exterior and we model the evaporation as a succession of states of decreasing mass, or (2) we use light-cone coordinates, in which the φ=0 singularity is visible and describe the evaporation process in terms of boundary dynamics.

Using the results of [43,44] as a guide, we will find that in both cases, the end point of the black hole evaporation process is a full, regular AdS${}_{2}$ space-time endowed with a constant (vanishing) dilaton, i.e., a singularity-free state with zero mass and zero entropy.

### 3.1. Black Hole Evaporation in the Static Patch

We consider for simplicity the evaporation of an initially static black hole of a given mass *M*. We model the evaporation process as a sequence of static states of decreasing mass, without considering matter fluxes for now, i.e., $\partial_{+}f_{i}=\partial_{-}f_{i}=0$, as in [43]. Following [43], if one neglects the backreaction, black hole evaporation can be described as a generalized Unruh effect [65]. Similarly to what happens in quantizing scalar fields in Rindler space-time, an AdS${}_{0}$ observer will detect the AdS${}_{+}$ vacuum as filled with a thermal flux of particles, with a Planckian spectrum at a temperature given by Equation (5). The main difference between Minkowski/Rindler and AdS${}_{0}$/AdS${}_{+}$ space-times is that, in the latter case, thermal effects are not related to a physical relative accelerated motion of different observers, but have purely topological origin [43].

The relevant equations are

(35) $$\left\{\begin{array}{cc} \; & \rho^{\prime \prime }=\frac{\Lambda^{2}}{4}e^{2\rho };\\ \; & \varphi^{\prime \prime }=\frac{\Lambda^{2}}{2}e^{2\rho }\varphi ;\\ \; & \varphi^{\prime \prime }-2\rho^{\prime }\varphi^{\prime }=\frac{N}{12}\left[{\left(\rho^{\prime }\right)}^{2}-\rho^{\prime \prime }\right], \end{array}\right.$$

where primes here stand for derivation with respect to the static coordinate $\sigma \equiv \frac{2}{a\Lambda }\mathrm{arcsinh}\left[{\left(\frac{\Lambda^{2}r^{2}}{a^{2}}-1\right)}^{-1/2}\right]$.

It is important to notice that the new coordinate −∞<σ⩽0 covers only the outside horizon region. In particular, the space-time singularity at φ=0 is not visible. Thus, in this coordinate system, the backreaction of the geometry cannot be described by the boundary dynamics and is encoded instead in the change of the parameter *a* (the black hole mass). A set of solutions of Equation (35) is given by

(36) $$\begin{array}{c} e^{2\rho }=\frac{a^{2}}{\sinh^{2}\left(\frac{a\Lambda }{2}\sigma \right)};\;\varphi =\frac{\phi_{0}a}{2}\coth\left(\frac{a\Lambda }{2}\sigma \right)+\frac{N}{24}\left[\frac{a\Lambda \sigma }{2}\coth\left(\frac{a\Lambda }{2}\sigma \right)-1\right], \end{array}$$

describing the AdS${}_{+}$ space-time, while

(37) $$\begin{array}{c} e^{2\rho }=\frac{4}{\Lambda^{2}\sigma^{2}};\;\varphi =\frac{\phi_{0}}{\Lambda \sigma }, \end{array}$$

corresponds to the AdS${}_{0}$ LDV written in terms of the coordinate σ.

One can easily check that when the black hole evaporates and *M* (hence the temperature (5)) decreases, the black hole interior region shrinks. When we take the zero mass limit, a→0, Equation (36) becomes Equation (37). At first glance, this seems to imply that the AdS${}_{+}$ black hole will settle down to the AdS${}_{0}$ vacuum at the end of evaporation. However, this is not actually true, being the CDV energetically preferred, according to the discussion of Section 2.1. A phase transition will bring the AdS${}_{0}$ LDV to the AdS${}_{2}$ CDV, with φ=0 (corresponding to ϕ=N/24). The end point of the black hole evaporation is therefore full AdS${}_{2}$, i.e., a regular space-time with zero mass and entropy. This strongly suggests that the evaporation of 2D AdS black holes is a unitary process.

### 3.2. Boundary Dynamics

In the previous subsection, we have used a coordinate system covering only the black hole exterior, in which the inner space-time boundary, i.e., the singularity, is not visible. Here, we use light-cone coordinates, which also cover the black hole interior and allow us to describe black hole evaporation in terms of boundary dynamics. We solve the field equations and the constraints, taking into account the contributions of the Hawking flux and the backreaction of the geometry. In the most general case, one should solve Equations (30)–(34) by considering a given profile of incoming matter, characterized by $T_{++}$, and impose appropriate boundary conditions to fix the functions $t_{\pm }$. Being the conformal factor of the metric fixed, this should allow finding the solution for $\varphi (x^{+},x^{-})$, which gives the boundary equation when equated to zero. However, for our purposes, we do not need to solve the equations in such cumbersome detail.

To keep the discussion as simple as possible, we do not consider the initial phase of black hole formation from incoming matter, but only the evaporation phase of an initially static black hole configuration of given mass *M*. Flux of Hawking radiation is switched on at time t=0 in a non-adiabatic way. This allows us to set $\partial_{+}f_{i}=0$ and $t_{+}=0$ in Equations (30)–(34) for t⩾0. Obviously, the form of the stress-energy tensor component $T_{--}$, which contains also information on the incoming matter, will be not determined in this way. We will therefore consider a general form for the stress-energy tensor $T_{--}$ of Hawking radiation, which will be described by a generic function of $x^{-}$ only, $T_{--}=\tau_{--}\left(x^{-}\right)$. We will then show, using quite general conditions on $\tau_{--}\left(x^{-}\right)$ that the SL(2, R) isometry of the metric can always be used to remove the future asymptotic timelike singularity of the space-time. We will then provide an explicit description of the whole evaporation process by choosing a particularly simple form for $\tau_{--}\left(x^{-}\right)$.

In order to solve the system (30)–(34) and provide a simple analysis of the dynamics of the boundary, it is convenient to introduce a function $M(x^{-},x^{+})$ parameterizing the field φ [61],

(38) $$\varphi =\frac{M(x^{-},x^{+})}{x^{-}-x^{+}}.$$

This allows us to rewrite Equations (30)–(34) as follows

(39) $$e^{2\rho }=\frac{4}{\Lambda^{2}}{\left(x^{-}-x^{+}\right)}^{-2};$$

(40) $$\left(x^{-}-x^{+}\right)\partial_{+}\partial_{-}M+\partial_{-}M---\partial_{+}M=0;$$

(41) $$\partial_{+}^{2}M=0;$$

(42) $$\partial_{-}^{2}M\left(x^{-},x^{+}\right)=-\left(x^{-}-x^{+}\right)\tau_{--}$$

where $\tau_{--}$ is the stress-energy tensor. As usual, the dynamics for the conformal factor decouples, so that we can solve the system (39)–(42) for M with ρ given by Equation (39). We obtain

(43) $$M\left(x^{-},x^{+}\right)=c_{1}+c_{2}\left(x^{+}+x^{-}\right)-\left(x^{-}-x^{+}\right)\;\int \int \;\tau_{--}dx^{-}+2\;\int \int \int \;\tau_{--}dx^{-}$$

where $c_{1,2}$ are integration constants. They can be fixed by requiring the solution for φ, given by Equation (38), to match the vacuum solution (20), when the Hawking flux is turned off ($\tau_{--}=0$). This gives $c_{1}=2\phi_{0}/\Lambda ,\;c_{2}=\phi_{0}a$ and the final form of the solution for φ is

(44) $$\varphi =\frac{1}{\left(x^{-}-x^{+}\right)}\left[\frac{2\phi_{0}}{\Lambda }+\phi_{0}a\left(x^{+}+x^{-}\right)-\left(x^{-}-x^{+}\right)\;\int \int \;\tau_{--}dx^{-}+2\;\int \int \int \;\tau_{--}dx^{-}\right].$$

At the end of Section 2.1, we have seen that the SL(2, R) isometry of the metric allows one to choose a light-cone frame in which the asymptotic observer does not see any singularity.

Let us now show that this is still true even in the presence of Hawking radiation and backreaction effects. For the full solution (44), the singularity trajectory φ=0 is described by:

(45) $$\frac{2\phi_{0}}{\Lambda }+\phi_{0}a\left(x^{+}+x^{-}\right)-\left(x^{-}-x^{+}\right)\;\int \int \;\tau_{--}dx^{-}+2\;\int \int \int \;\tau_{--}dx^{-}=0.$$

On the asymptotic timelike boundary $x^{-}=x^{+}$, we have

(46) $$x^{-}=-\frac{1}{\phi_{0}a}\left[\frac{\phi_{0}}{\Lambda }+\;\int \int \int \;\tau_{--}dx^{-}\right].$$

When $\tau_{--}$ is a positive-definite function, as it should be in the case of Hawking radiation, the right-hand side of Equation (46) is always negative. This implies, again, that we can always remove the future singularity in the asymptotic timelike boundary using an appropriate light-cone frame.

In order to have an explicit description of the black hole evaporation process, let us now model it in a simple way, as a sequence of steps in which the Hawking flux can be taken as constant. This allows us to fix $t_{-}=\mathrm{constant}$ in Equation (33), which is given in terms of a running black hole mass $\hat{M}⩽M$. $\hat{M}$ will decrease during the evaporation process, with $\hat{M}\to 0$ as the end point approaches.

It is well known that the stress-energy tensor describing the outgoing Hawking radiation is given in terms of the Schwarzian derivative of the static coordinate transformations (17) connecting the Schwarzschild and the conformal gauges, and reads [43]:

(47) $$\begin{array}{c} \langle 0|T_{--}|0\rangle =\frac{N}{48}a^{2}\Lambda^{2}. \end{array}$$

This equation determines the function $\tau_{--}$ in Equation (44) and allows writing explicitly the singularity Equation (45)

(48) $$\begin{array}{cc} \; & \frac{2\phi_{0}}{\Lambda \alpha^{2}}+\sqrt{\frac{2\hat{M}\phi_{0}}{\Lambda }}\left(x^{+}+x^{-}\right)-\left(x^{-}-x^{+}\right)\frac{N\hat{M}\Lambda }{24\phi_{0}}\left(\frac{{\left(x^{-}\right)}^{2}}{2}+C_{0}x^{-}+C_{1}\right)+\\ \; & \frac{N\hat{M}\Lambda }{12\phi_{0}}\left(C_{2}+C_{1}x^{-}+C_{0}\frac{{\left(x^{-}\right)}^{2}}{2}+\frac{{\left(x^{-}\right)}^{3}}{6}\right)=0, \end{array}$$

where we used the expression of the (running) ADM mass, i.e., $\hat{M}=\Lambda \phi_{0}a^{2}/2$, while the $C_{i}$s are integration constants. From Equation (47), it immediately follows

(49) $$C_{2}+C_{1}x^{-}+C_{0}\frac{{\left(x^{-}\right)}^{2}}{2}+\frac{{\left(x^{-}\right)}^{3}}{6}>0.$$

Evaluating Equation (48) on the asymptotic boundary $x^{+}=x^{-}$, we obtain

(50) $$\begin{array}{c} f\left(x^{-}\right)\equiv \frac{2\phi_{0}}{\Lambda \alpha^{2}}+2\sqrt{\frac{2\hat{M}\phi_{0}}{\Lambda }}x^{-}+\frac{N\hat{M}\Lambda }{12\phi_{0}}\left(C_{2}+C_{1}x^{-}+C_{0}\frac{{\left(x^{-}\right)}^{2}}{2}+\frac{{\left(x^{-}\right)}^{3}}{6}\right)=0. \end{array}$$

As expected, the function $f(x^{-})$ is positive definite so that there is no future asymptotic singularity.

It is important to stress that $\hat{M}$ becomes smaller and smaller as the evaporation proceeds. In our simplified picture, the apparent black hole horizon shrinks as the evaporation proceeds and the asymptotic timelike observer will never hit the singularity. At the end of evaporation, we have $\hat{M}=0$ and the Hawking flux also vanishes. From Equation (48) follows that the space-time boundary disappears, i.e., the solution becomes the LDV solution (18). This also holds true for the general solution (44). $\tau_{--}$ must be a monotonically decreasing function of $x^{-}$ and a→0 at the end point of the evaporation process, implying that solution (44) becomes the LDV given by Equation (18).

One should keep in mind that, although the asymptotic observer does not encounter any future singularity during the evaporation process, the AdS${}_{0}$ space-time is not geodesically complete, as can be seen by the fact that radial null geodesics suddenly terminate at finite length at r→0, i.e., at the space-time singularity ϕ=0 (see Equation (3)), which prevents them from being continued beyond [66]. This is of course due to the fact that we cut the space-time at r=0 (see Section 2.1). Only its maximal extension, i.e., the full AdS${}_{2}$ space-time, is singularity free and geodesically complete. On the other hand, the stability argument of [44] can be used again to argue that the true end point of the evaporation process will be the CDV with $\phi_{0}=0$, full AdS${}_{2}$ space-time, consistent with unitarity.

In the next sections, we will confirm this conclusion by investigating the evolution of the entanglement entropy of Hawking radiation during evaporation and by constructing its Page curve.

## 4. Entanglement Entropy of 2D AdS Black Holes

In the present and the following sections, we tackle the information problem during the evaporation process of a 2D JT black hole. We will do this by taking into account the entanglement entropy of both black hole and Hawking radiation. The peculiarities of 2D AdS gravity will allow us to have a precise, quantitative description of the EE of the hole and of Hawking radiation during the entire evaporation process, which accounts for the information flow between them.

In two space-time dimensions, black hole entropy can be fully ascribed to quantum entanglement. This is due to the fact that the 2D Newton constant (parameterized by the dilaton) is wholly induced by quantum fluctuations of the geometry. This can be shown by working in the AdS/CFT correspondence framework, which peculiarly has a non-holographic realization in two space-time dimensions in terms of a dual (chiral) two-dimensional CFT (CFT${}_{2}$) living in the bulk [67,68]. The existence of this dual quantum gravity theory is a crucial ingredient because it allows computing the EE of the JT black hole in terms of the EE of the dual CFT${}_{2}$ in the curved gravitational background [45]

(51) $$S_{ent}^{\left(bh\right)}=\frac{c}{6}\ln\left(\frac{L}{\pi r_{h}}\sinh\frac{\pi r_{h}}{L}\right),$$

where $c=12\phi_{0}$ is the central charge of the CFT ($\phi_{0}$ plays the role of the 2D inverse Newton constant) and *L*, $r_{h}$ are the AdS length (related to the inverse of the cosmological constant, i.e., $L=\Lambda^{-1}$) and the black hole radius, respectively. Notice that Equation (51) holds true only when we are allowed to use our effective description of AdS${}_{2}$ quantum gravity in terms of the dual CFT${}_{2}$ with c≫1, corresponding to the weakly-coupled regime of the gravitational theory, $\phi_{0}^{-1}\ll 1$.

The computations of [45], leading to Equation (51), are performed using an Euclidean instanton and can therefore be easily extended to the case of the AdS${}_{-}$, i.e., a black hole with “negative mass”. In the following, we will make use of this result to describe the EE of the evaporating black hole interior in a simple way.

The thermal, Bekenstein–Hawking (BH) entropy $S_{BH}$ of AdS${}_{2}$ black holes can be derived as the leading term in the large mass expansion of the EE (51). In fact, for large black holes, $r_{h}/L\gg 1$, $S_{ent}^{\left(bh\right)}$ gives the BH entropy with a subleading log term:

(52) $$\begin{array}{c} S_{ent}^{\left(bh\right)}\approx S_{BH}-2\phi_{0}\ln S_{BH}. \end{array}$$

Equation (51) describes the entanglement entropy of an eternal AdS black hole and is in agreement with several results, which appeared in the literature:

- Classical space-time structure, in particular its connectedness, emerges out of quantum entanglement [48,49,50];
- The BH entropy has its origin in the entanglement entropy of the two edges of maximally extended AdS${}_{2}$ space-time when the degrees of freedom (DOF) in one edge are traced out [69]. The result (51) is a slightly different realization of the idea proposed in [69], where the entanglement entropy is generated by two copies of a CFT in an initial entangled state and by using a thermo-field double. Equation (51) is instead obtained using a single CFT defined in the maximally extended space-time by tracing the degrees of freedom (DOF) in half of it;
- Holographic entanglement entropy formulas [33,46,47] give the EE of maximally extended AdS space-time in terms of the area of co-dimension two minimal surfaces, which can be identified with the event horizon.

Altogether, these results indicate that black holes are quantum gravity objects (possibly at horizon scale) for which the relevant DOF are not localized near the event horizon, as a simple-minded interpretation of the Bekenstein–Hawking area law would suggest. The horizon area dependence of the BH entropy, and hence its holographic nature, should be therefore related to the area scaling law of EE in QFT.

Equation (51), together with the microscopic derivation of the BH formula given in [21], gives a simple and intuitive characterization of the information content of an eternal JT black hole. As shown in [21], the BH entropy simply counts the microstates of the CFT${}_{2}$ which are dual to the black hole of mass *M*, whereas Equation (51) tells us that information is stored in the black hole in the form of quantum correlations localized in the black hole interior. On the other hand, Equation (51) does not reveal if and how the information stored in the black hole comes out during evaporation. In order to understand this aspect, we need to discuss the Page curve and the information flow for the evaporating JT black hole. This will be the subject of the next section.

## 5. Information Flow and the Page Curve for Evaporating JT Black Holes

Let us now consider the black hole evaporation process in terms of the information flow between the shrinking black hole interior and its exterior, and the information carried by Hawking radiation. We will describe this in a quantitative way, by computing the EE of the black hole with its exterior and the EE of Hawking radiation. We will use a simplified description of the process in terms of a sequence of static states characterized by constant black hole radius $r_{h}$, so that the black hole EE can be given as a function of $r_{h}$ at any time.

As seen in the previous sections, this simplified picture is fully justified in the JT gravity context, since the evaporation process, including backreaction can be described as a sequence of static states (see Section 3.2). We will also consider the simplest case in which the Hawking radiation is given by *N* right-moving species of 2D massless scalar fields $f_{i}$. This will result in a particularly symmetric situation in which Hawking radiation is treated in the same way as AdS${}_{2}$ quantum gravity, i.e., a (chiral) CFT${}_{2}$ with central charge c=N. Although this may not be the most general situation, it is simple enough to tackle the conceptual puzzles involved in the black hole evaporation.

### 5.1. Hawking Radiation

In order to describe Hawking radiation, we parameterize the 2D black hole geometry using, as usual, two sets of light-cone coordinates defined in the previous sections. The coordinates u=t+r,v=t−r, pertaining to the frame of the asymptotic observer, are expressed in terms of its time *t* and radial coordinate *r* and cover the black hole exterior only. The coordinates $U=x^{+},\;V=x^{-}$ define, instead, the frame of the inertial observer falling through the black hole horizon, with a related time coordinate $\tau =(x^{+}+x^{-})/2$. Correspondingly, the asymptotic observer will expand quantum fields using $b_{\nu }$, $b_{\nu }^{†}$ modes of a given *t*-frequency ν, whereas the infalling observer will use $a_{\mu }$, $a_{\mu }^{†}$ modes of a given τ-frequency μ. The modes $b_{\nu }$ can be expressed in terms of $a_{\mu }$, $a_{\mu }^{†}$ by a Bogoliubov transformation [1,4,70]. In this way the *a*-vacuum ${|a\rangle }_{a}$ (the quantum vacuum for the infalling observer) is seen as a bath with a thermal spectrum, at the black hole Hawking temperature $T_{H}$, by the asymptotic observer.

The modes $b_{\nu }$ are not enough to calculate the EE of Hawking radiation, as they are only defined in the outside region (region *I*). Since Hawking radiation is entangled with the black hole interior, we need to introduce the quantum field modes ${\hat{b}}_{\nu }$, which are defined in the black hole interior (region II) [1,4]. Denoting with *H* the Hamiltonian of the full system, the mode $b_{\nu }^{†}$ raises the energy by a quantum ν: $\left[H,b_{\nu }^{†}\right]=\nu b_{\nu }^{†}$ (i.e., it creates a particle of energy ν in the outside region *I*), whereas ${\hat{b}}_{\nu }^{†}$ lowers the energy by a quantum ν: $\left[H,{\hat{b}}_{\nu }^{†}\right]=-\nu {\hat{b}}_{\nu }^{†}$ (i.e., it creates a particle of negative energy −ν in the interior region II). One can easily show that the *a*-vacuum can be expressed in terms of $b_{\nu }^{†}$ and ${\hat{b}}_{\nu }^{†}$ as [4]

(53) $${|a\rangle }_{a}=A\exp\left(\int \frac{d\nu }{2\pi }e^{-\nu /T_{H}}b_{\nu }^{†}{\hat{b}}_{\nu }^{†}\right){|0\rangle }_{b,\hat{b}},$$

with A normalization factor and ${|0\rangle }_{b,\hat{b}}$ the *b*-vacuum. This equation tells us that the *b*-modes of Hawking radiation are entangled with the $\hat{b}$ modes in the black hole interior.

### 5.2. The Page Curve

During the evaporation process, the thermal entropy of a JT black hole of initial radius $r_{h}=R_{H}$ and final radius $r_{h}=0$ decreases from the initial value $S_{BH}\left(R_{H}\right)=2\pi \phi_{0}\Lambda R_{H}$ to the final $S_{BH}\left(0\right)=0$. Correspondingly, the thermal entropy of the Hawking radiation $S_{R}$, which is roughly proportional to the number of quanta emitted, will grow from $S_{R}=0$ to $S_{R}\approx M_{BH}/T_{H}\approx S_{BH}\left(R_{H}\right)$. This is the essence of the information loss problem: assuming the JT black hole is formed by the collapse of a quantum pure state, the evaporation process transforms a pure into a mixed quantum state.

Conversely, if we assume that evolution is unitary or, more precisely, if we assume the validity of the so-called “Central Dogma” [22], the quantum state of Hawking radiation has to be purified, and so its EE $S_{E}$ must go to zero in the final stages of the evaporation process.

Although there is no general consensus about the mechanism that purifies the radiation, Page has shown, using general principles of information theory, that information can only come out at late times. The result is the famous Page curve for $S_{E}$ [29,30]. $S_{E}$ starts from zero and initially grows, closely following the thermal entropy of the radiation $S_{R}$, until the latter intersects $S_{BH}$ at approximately half-way of the evaporation process. At the intersection point $t=t_{Page}$, called Page time, $S_{E}$ reaches a maximum and then decreases, closely following the Bekenstein–Hawking entropy curve $S_{BH}$ at late times. At the end of the evaporation, $S_{E}$ becomes zero again (the final state is a pure state).

### 5.3. 2D Black Hole Information

A crucial issue in explaining the Page curve is to understand the way information can flow from the black hole into late Hawking radiation, so that the radiation final state can be purified. Recent attempts, such as the wormhole and the island proposal (see, e.g., [22,31,32,51,54,55,56,57,58,59,60]) have been focused on effects of the (Euclidean) low-energy gravitational theory, which may be responsible for transferring information from the evaporating black hole interior to the outside radiation. This latter approach is not completely satisfactory because it leaves the question about the microscopic origin of these low-energy effects unanswered. Despite some interesting proposals (see, e.g., [23,24,25,27,28,71,72,73,74,75,76]), we are far away from having a clear hint of how the $N∼S_{BH}$ quantum states building the black hole may evolve unitarily during black hole evaporation, transferring completely the information of the initial pure state, which collapsed to form the black hole, into late time Hawking radiation.

The strategy we follow in this paper is to use a simplified model for black hole evaporation, which allows connecting the microscopic effective description of the black hole in terms of a 2D CFT with the evaporation process. This is possible on account of the simplicity and peculiarities of 2D JT gravity. In fact, it has been observed that the black hole entropy can be fully ascribed to quantum entanglement if the Newton constant is induced by quantum fluctuations [77]. The original version of the proposal referred to quantum fluctuations of matter fields, but, in the context of the AdS/CFT correspondence in 2D, it has been extended to the CFT degrees of freedom dual to 2D AdS gravity. The peculiarity of the AdS/CFT correspondence in 2D is the fact that it has a bulk/bulk realization, in terms of a chiral CFT living in 2D [21,67,68]. It follows that thermodynamics and the evaporation process of the JT black hole allow for an effective description in terms of a 2D CFT with central charge *c* given by the inverse of 2D Newton constant $\phi_{0}$ [21]:

(54) $$c=12\phi_{0}.$$

In particular, this means that black hole entropy has its origin in the quantum entanglement of *c* microscopic DOF, which gives an effective description of AdS${}_{2}$ quantum gravity. $S_{ent}^{\left(bh\right)}$ equals the thermal entropy $S_{BH}$ for large black holes, i.e., when thermal fluctuations dominate. In this regime, we expect a semiclassical description to hold, i.e., the black hole to be described by a quantum, thermal, CFT of central charge *c* in a classical gravitational background endowed with an event horizon. Away from this semiclassical regime, we have contributions coming from the quantum entanglement of the microscopic DOF. As expected, these corrections are negative (see Equation (52)). In this generic quantum gravity regime, we cannot simply describe the system as a QFT in a fixed background geometry endowed with an event horizon. We expect quantum contributions to the geometry to become relevant, the classical notion of horizon to loose much of its meaning and the inner structure of the black hole to play a role in the black hole information problem.

These features are also evident in the computations of [45] leading to Equation (51). The EE is calculated using an Euclidean instanton and it arises, similarly to [69], by tracing out the CFT degrees of freedom over part of the space. In this description, the BH entropy is not simply the Boltzmann entropy of a CFT living in a boundary (the stretched horizon of the black hole) of a structureless interior black hole space-time, but it is rather due to quantum entanglement of the black hole interior with the outside world. This change of perspective also implies that the holographic nature of the Bekenstein–Hawking formula has its roots in the scaling of the EE with the area of the boundary separating the observable from the unobservable region.

The interpretation of the black hole entropy as the semiclassical limit of the EE of some microscopic quantum gravity (QG) DOF localized in the black hole interior is also consistent with the proposal of [78], which sees black hole thermodynamics as a manifestation of long-range quantum gravity effects. In particular, the (generalized) thermal equivalence principle (GTEP) [79], which is used to explain the Hawking temperature, should be seen as a universal property of semiclassical horizons in the sense explained above.

Presently, we do not have a precise formulation of AdS${}_{2}$ quantum gravity, but only a low-energy effective description in terms of a 2D CFT with central charge given by Equation (54). We will therefore use an extremely simplified model to investigate the implications of our quantum entanglement-based description for the black hole information problem. Our discussion will remain quite general and based on general principles of QFT, so that we will not need to know an exact formulation of AdS${}_{2}$ quantum gravity. We will merely assume that such a formulation does indeed exist.

### 5.4. Entanglement Entropy of Hawking Radiation

In our simplified model, we describe black hole evaporation as the emission of Hawking radiation (the modes $b_{\nu }$ of positive frequency ν) in the exterior region together with the formation of a state of negative mass (the modes ${\hat{b}}_{\nu }$ of negative frequency −ν) in the black hole interior. We have already seen that the two sets of modes are entangled and can be considered as two subsets, $H_{b}$, $H_{\hat{b}}$ of the full Hilbert space spanned by the modes *a*. The EE of the radiation $S_{E}$ is the von Neumann entropy obtained by partial tracing the full density matrix $\rho_{b\hat{b}}$ over $H_{\hat{b}}$. Well-known properties of the EE imply that $S_{E}$ can be also calculated by partial tracing the density matrix over $H_{b}$

(55) $$S_{E}=-Tr_{\hat{b}}\rho_{\hat{b}}\ln\rho_{\hat{b}},$$

where $\rho_{\hat{b}}:=Tr_{b}\rho_{b\hat{b}}$.

In order to simplify the calculation of $S_{E}$, we will assume that all the modes of negative frequency −ν will be localized in a connected space-like slice of size $\Sigma ⩽R_{H}$ of the black hole interior, where $R_{H}$ is the initial black hole radius. This is not necessarily true. We could also have contributions coming from several disconnected regions—islands using the terminology of [22,31,32,55,56]. However, we do not expect this contribution to change at least the qualitative behavior of our final results. With this assumption, we consider the localized, connected structure generated by clumping the $\hat{b}$-modes of negative energy as a 2D black hole of negative energy in AdS${}_{2}$, with Σ playing the role of the radius of the negative mass black hole. The entanglement entropy of an AdS${}_{2}$ black hole with negative mass has been calculated in [45]. Using this result, we obtain

(56) $$S_{E}=\frac{N}{6}\ln\left(\frac{L}{\pi \delta }\sin\frac{\pi \Sigma }{L}\right),$$

where *N* is the number of species of fields and L, δ are the IR, UV cutoffs respectively. Being $\Sigma ⩽R_{H}$, we can identify the IR cutoff as the initial black hole radius: $L=R_{H}$. Let us now denote with $r_{h}$ the time-dependent value of the black hole horizon during evaporation. $r_{h}$ runs from $r_{h}=R_{H}$ at the beginning of evaporation to $r_{h}=0$ at the end. Correspondingly, $\Sigma =R_{H}-r_{h}$ runs from 0 to $R_{H}$. We get therefore for the EE entropies of the black hole and Hawking radiation

(57) $$S_{E}\left(r_{h}\right)=\frac{N}{6}\ln\left[\frac{R_{H}}{\pi \delta }\sin\frac{\pi (R_{H}-r_{h})}{R_{H}}\right],\;S_{ent}^{\left(bh\right)}\left(r_{h}\right)=\frac{c}{6}\ln\left(\frac{\delta }{\pi r_{h}}\sinh\frac{\pi r_{h}}{\delta }\right),$$

where we have used the fact that the UV cutoff is of order *L* (see [45]) and set δ=L in Equation (51).

The entanglement entropy (57) has well-known UV divergences caused by the contribution of arbitrarily short wavelength modes. In our case, these divergences manifest themselves at the beginning ($r_{h}=R_{H}$) and end point ($r_{h}=0$) of evaporation, when $S_{E}$ blows up. At these points, we have the leading logarithmic divergences

(58) $$S_{E}≃\frac{N}{6}\ln\frac{r_{h}}{\delta },\;S_{E}≃\frac{N}{6}\ln\left(\frac{R_{H}-r_{h}}{\delta }\right).$$

**Figure 2:**
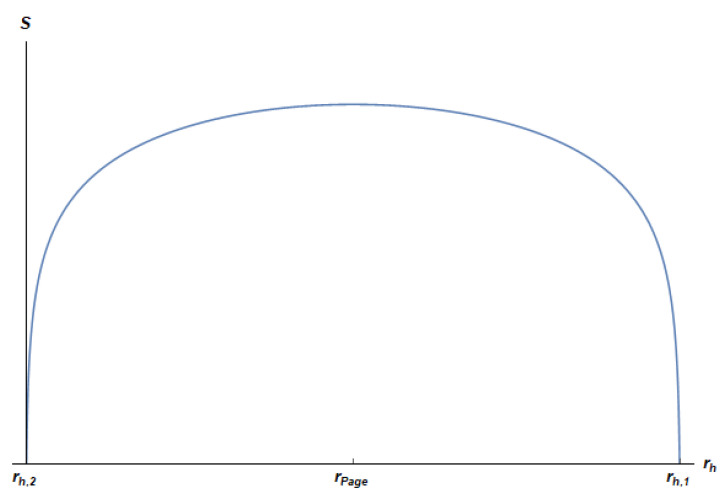
Regularized entanglement entropy $S_{E}^{\left(reg\right)}$ of the radiation as a function of the black hole radius $r_{h}$, for the following selected values of the parameters: N=1 and $R_{H}/\delta =1000$. We plot $S_{E}$ for $\delta \approx r_{h,2}⩽r_{h}⩽r_{h,1}\approx R_{H}-\delta$. $S_{E}$ starts from zero at $r_{h}\approx \delta$, reaches its maximum at the Page radius $r_{h}=r_{Page}=R_{H}/2$ when the black hole has reduced its size by a factor 1/2, then decreases down to zero at the end of the evaporation process, at $r_{h}\approx R_{H}-\delta$. This behavior is consistent with a unitary evaporation process and has the form of the Page curve. Note that time runs towards decreasing values of $r_{h}$.

The UV regulator δ of the dual CFT can be used to remove these divergences just by cutting off $r_{h}$ at distances above $R_{H}-\delta$ and below δ. The regularized EE $S_{E}^{\left(reg\right)}$ can then be obtained just by cutting the curve at the points $r_{h,2}=\frac{R_{H}}{\pi }\arcsin\left(\frac{\pi \delta }{R_{H}}\right)$ and $r_{h,1}=\frac{R_{H}}{\pi }\left[\pi -\arcsin\left(\frac{\pi \delta }{R_{H}}\right)\right]$ where $S_{E}$ vanishes (see Figure 2). For small values of $\delta /R_{H}$, the intersection points become $r_{h,2}\approx \delta$ and $r_{h,1}\approx R_{H}-\delta$.

Alternatively, we can subtract the finite $S_{E}\left(r_{h}=\delta \right)$ term from $S_{E}$ in such a way that the regularized EE of radiation $S_{E}^{\left(reg\right)}=S_{E}\left(r_{h}\right)---S_{E}\left(r_{h}=\delta \right)$ always vanishes exactly at $r_{h}=\delta$ and $r_{h}=R_{H}-\delta$. Notice that the term we are subtracting $S_{E}\left(r_{h}=\delta \right)=\frac{N}{6}\ln\left(\frac{R_{H}}{\pi \delta }\sin\frac{\pi \delta }{R_{H}}\right)$ consistently vanishes in the limit $\delta /R_{H}\to 0$.

#### 5.4.1. Thermal Entropy of Hawking Radiation and Relation between *N* and *c*

The thermal entropy $S_{R}$ of Hawking radiation gives an upper bound for its entanglement entropy $S_{E}$ and characterizes the thermodynamical regime of the radiation, where thermal correlations dominate over the quantum ones. This allows for a coarse grained description of the radiation in terms of a thermal density matrix. The computation of $S_{R}$ and the use of simple thermodynamical arguments will also allow us to find a relation between the central charge *c* of the CFT describing the JT black hole and the number of field species *N* in the Hawking radiation.

We derive the thermal entropy of the Hawking radiation at large temperature by first computing the von Neumann entropy of a single mode in a thermal bath at temperature *T* and then integrating over the total number of modes. We put the system in a 1D box of finite size *ℓ*, which therefore acts as an IR cutoff. The spectrum for the eigenvalues $E_{m}$ of the Hamiltonian of the system will be therefore discrete. At large *T*, we can take $E_{m}=m\omega$, where *m* is a (positive) integer and ω is of order 1/ℓ.

The state (53) corresponds to a thermal density matrix ρ of a single mode

(59) $$\rho_{mm^{\prime }}=\delta_{mm^{\prime }}\frac{e^{-\beta E_{m}}}{Z},$$

where β=1/T, with *T* temperature of the thermal bath in thermal equilibrium with the hole. Z is the partition function

(60) $$Z=\mathrm{Tr}\left(e^{-\beta \hat{H}}\right)=\sum_{m=0}^{\infty }e^{-m\omega /T}=\frac{e^{\omega /T}}{e^{\omega /T}-1}={\left(1-e^{-\omega /T}\right)}^{-1},$$

where $\hat{H}$ is the Hamiltonian of the system. Since we are considering T≫0, we can neglect the contribution of the vacuum.

The normalized eigenvalues of the density matrix (59) thus are:

(61) $$p_{m}=\left(1-e^{-\omega /T}\right)e^{-m\omega /T}.$$

It is easy to check that the normalization condition Trρ=1 is satisfied. The corresponding von Neumann entropy of the mode is:

(62) $$\begin{array}{cc} S_{\omega } & =-\sum_{m=0}^{\infty }p_{m}\ln p_{m}=\frac{\omega /T}{e^{\omega /T}-1}-\ln\left(1-e^{-\omega /T}\right). \end{array}$$

To compute the total entropy, we need to integrate over the number of modes N(ω). If we consider the 1D volume *ℓ*, each mode has wavenumber k=mπ/ℓ, where *m* is again a positive integer. However, k=ω, so m=ωℓ/π. The total number of modes in the *m*-space is $N=N\frac{1}{2}\cdot 2m=Nm$, where 2m is the volume of the 1D box, the factor 1/2 accounts for the fact that *m* is positive and *N* for the number of species of fields.

In terms of ω, $N\left(\omega \right)=N\frac{\ell }{\pi }\omega$, and thus, we have

(63) $$\begin{array}{cc} S_{R} & =\int S_{\omega }\;dN\left(\omega \right)=N\frac{\ell }{\pi }\int_{0}^{\infty }\left[\frac{\omega /T}{e^{\omega /T}-1}-\ln\left(1-e^{-\omega /T}\right)\right]d\omega =N\frac{\pi }{3}T\;\ell . \end{array}$$

As expected, the coarse-grained thermal entropy of the Hawking radiation is given by the thermal entropy for a 2D CFT (massless bosons) on the plane with IR regulator *ℓ* and central charge given by:

(64) $$c_{HR}=N.$$

As Hawking radiation lives in the AdS${}_{2}$ background, the most natural choice for the IR regulator *ℓ* is to be of the order of the AdS length *L*. In the following, we will therefore set ℓ=πL.

At first sight, the use of *L* both as the UV regulator δ of the CFT dual to AdS${}_{2}$ quantum gravity and as the IR regulator *ℓ* for Hawking radiation in the AdS${}_{2}$ background seems contradictory. This apparent contradiction can be solved by the UV/IR connection in the context of the AdS/CFT correspondence [80], which relates the IR cutoff of the gravity theory with the UV cutoff of the dual CFT.

So far, the central charge (54) of the CFT describing AdS${}_{2}$ quantum gravity and the central charge (64) of the CFT describing the Hawking radiation are two completely independent quantities. Let us now use a standard thermodynamical argument to show that $c=c_{HR}$. Let us consider the emission of an infinitesimal amount of energy $dE_{q}$ from a black hole of radius $r_{h}$ and corresponding mass *M* and temperature $T_{H}$ given by Equations (4) and (5) as a reversible process. After this emission, the black hole mass decreases to $M-dE_{q}$, its radius decreases to $r_{h}-dr_{h}$, whereas the black hole entropy decreases by the amount $dS_{q}\equiv \frac{dE_{q}}{T_{H}}$. Conservation of energy implies:

(65) $$\frac{\phi_{0}}{2L^{3}}{\left(r_{h}-dr_{h}\right)}^{2}+dE_{q}=\frac{\phi_{0}}{2L^{3}}r_{h}^{2}.$$

Neglecting terms of order $O\left(dr_{h}^{2}\right)$ and using Equation (5) for the black hole temperature, we have

(66) $$dE_{q}=\frac{\phi_{0}}{L^{3}}{\left(2\pi L^{2}\right)}^{2}T_{H}\;dT_{H}.$$

This gives the entropy change for the hole:

(67) $$dS_{q}=\frac{dE_{q}}{T}=4\pi^{2}\phi_{0}L\;dT_{H}.$$

Equating this change of thermal entropy with that of radiation, given by Equation (63), we obtain

(68) $$N=12\phi_{0}=c,$$

where we used Equation (54). This is a non-trivial relation between the number of species of fields *N* in the Hawking radiation and the central charge *c* of the CFT dual to the AdS${}_{2}$ quantum gravity. The result is a consequence of the interplay between thermodynamics and field-theoretical features (in particular the AdS/CFT correspondence). Specifically, it follows from (1) the reversible transfer of coarse grained entropy from the black hole to the radiation [81,82] $dS_{BH}=-dS_{R}$, (2) the description of both the black hole and the Hawking radiation in terms of a 2D CFT and the related linear scaling (63) of the entropy with the temperature, which allows writing the number of species *N* in the Hawking radiation in terms of the (inverse of) 2D Newton constant $\phi_{0}$ and (3) the AdS/CFT correspondence which implies Equation (54), i.e., it allows writing the central charge of the CFT dual to AdS${}_{2}$ gravity in terms of $\phi_{0}$.

It is also interesting to notice that the derivation above is fully consistent with a corpuscular description of the black hole [78], i.e., its description in terms of a bound state of *n* quanta of N=c number of species, with energy of the order of the temperature of the black hole, i.e., $E_{q}∼T=r_{h}/2\pi L^{2}$. The corresponding infinitesimal change of the black hole mass when *n* quanta are emitted is therefore $dM∼E_{q}dn\Rightarrow dn∼dM/E_{q}∼\frac{2\pi L^{2}}{r_{h}}\;dM$. However, $dM∼\frac{N}{12L^{3}}r_{h}\;dr_{h}$, and hence:

(69) $$dn∼\frac{\pi }{6L}Ndr_{h}\Rightarrow n∼\frac{\pi }{6L}Nr_{h}=S_{BH}.$$

In the corpuscular description, Hawking evaporation is just the transfer of energy and entropy from *n* quanta building the black hole to the *n* Hawking radiation quanta. Again, energy and entropy conservation require the number of species building the black hole to be the same of that composing Hawking radiation.

#### 5.4.2. Entanglement Entropy and Page Curve for the JT Black Hole

In the previous sections, we have computed all quantities characterizing the black hole and Hawking radiation, which are relevant from the point of view of quantum information and thermodynamics, namely $S_{BH}$ (Equation (6)), $S_{R}$ (Equation (63)), $S_{E}^{\left(reg\right)}$ (left expression in Equation (57))

(70a) $$\begin{array}{cc} \; & S_{R}=\frac{\pi c}{6}\frac{R_{H}-r_{h}}{\delta }; \end{array}$$

(70b) $$\begin{array}{cc} \; & S_{E}^{\left(reg\right)}=\frac{c}{6}\ln\left(\frac{R_{H}}{\pi \delta }\sin\frac{\pi r_{h}}{R_{H}}\right); \end{array}$$

(70c) $$\begin{array}{cc} \; & S_{BH}=\frac{\pi c}{6}\frac{r_{h}}{\delta }. \end{array}$$

We can now plot and discuss the Page curve for the JT black hole.

**Figure 3:**
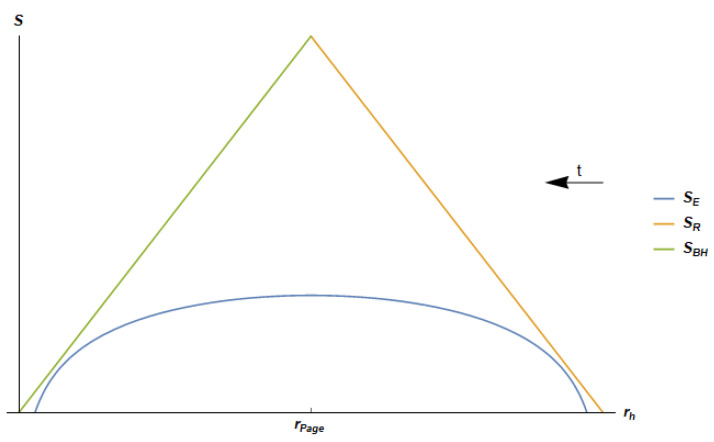
Qualitative plot of the entanglement entropy $S_{E}^{\left(reg\right)}$ of the radiation, thermal entropy of the radiation $S_{R}$ and the Bekenstein–Hawking entropy $S_{BH}$ as a function of $r_{h}$. We show the curves for the following selected values of the parameters: $R_{H}=100$, N=1 and δ=20. As δ approaches zero, the two zeros of $S_{E}^{\left(reg\right)}$ approaches to $r_{h}=0$ and $r_{h}=R_{H}$. Note that the time runs towards decreasing values of $r_{h}$.

The Page curve for the JT black hole is shown in Figure 3, where it is compared with the thermal entropy of Hawking radiation and to the Bekenstein–Hawking entropy of the black hole. Note that time increases towards decreasing values of $r_{h}$, from the beginning t=0 to the ending $t=t_{E}$ of evaporation. This corresponds to $r_{h}$ decreasing from the initial regularized black hole radius $r_{h}=R_{H}-\delta$ to the final regularized radius $r_{h}=\delta$. The plots show a behavior of $S_{E}^{reg}$ which is fully consistent with unitary evolution during the evaporation process and has the form of the Page curve. The EE of radiation starts from zero at $r_{h}=\delta$, reaches its maximum when the black hole has reduced its size by a factor 1/2, $r_{h}=R_{H}/2$, then decreases monotonically down to zero at the end of the evaporation process, at $r_{h}=R_{H}-\delta$.

Conversely, the EE of the black hole, which for large ($r_{h}\gg L$) black hole is well approximated by the thermodynamical entropy $S_{BH}$, starts from its maximum value at $r_{h}=R_{H}$, then monotonically decreases to zero at $r_{h}=0$.

The Bekenstein–Hawking entropy $S_{BH}$ and the thermal entropy of radiation $S_{R}$ (green and orange solid lines, respectively) behave linearly as a consequence of the CFT description and of the space-time dimensionality. The regularized entanglement entropy $S_{E}^{\left(reg\right)}$ closely tracks the behavior of the thermal entropy of the radiation $S_{R}$ and the Bekenstein–Hawking $S_{BH}$ curves at the beginning and at the end of evaporation, respectively, while in the other stages, it is determined by the behavior of the lnsin function. This is because, initially, most of the correlations in the radiation have thermal nature. As the evaporation proceeds, $S_{E}^{\left(reg\right)}$ begins to deviate strongly from $S_{R}$. At half evaporation, at the Page time, $r_{h}=R_{H}/2$ in our 2D case, $S_{E}$ reaches its maximum and the correlations in the radiation begin to extract information from the black hole. At the end of the evaporation process, when most of the information has been extracted from the hole, $S_{E}$ catches up with $S_{BH}$ and becomes thermal again.

The curve for $S_{E}^{\left(reg\right)}$ is symmetric with respect to the “Page radius” $R_{H}/2$. This seems, again, to be a consequence of the low D=2 space-time dimensionality.

It is important to stress again that, thanks to the equality between *N* and the central charge *c* of the CFT dual to AdS${}_{2}$ gravity, Equation (68), the $S_{E}$ curve is always bounded from above by the coarse-grained entropies $S_{R}$ and $S_{BH}$. As in Page’s argument [29], this is due to the fact that the latter two retain less information, being related to thermal states, representing thus the upper bound of the entanglement entropy.

## 6. Conclusions

In this paper, we have investigated the semiclassical dynamics of 2D dilatonic JT black holes and derived the Page curve for their entanglement entropy. Our results are fully consistent with unitarity of the evaporation process. Specifically, we have shown that the end point of the evaporation is 2D AdS space-time with vanishing dilaton—a perfectly regular state with zero mass and entropy. We have also shown that, during the evaporation, the behavior of the entanglement entropy of the radiation agrees with Page’s argument and with information preservation. In fact, the EE of the radiation initially grows, following a thermal behavior, reaches a maximum at half-way of the process, and then goes down to zero, following the Bekenstein–Hawking entropy of the black hole. Moreover, the existence of a dual CFT description for the JT black hole and usual thermodynamic arguments imply a non-trivial identification of the central charge of the CFT dual to AdS${}_{2}$ gravity with the number of species of fields in the Hawking radiation.

We have used a simplified model to discuss the semiclassical dynamics and to describe the black hole interior. In particular, we have modeled the evaporation process by considering the contribution of a single, connected configuration of negative mass—or island, forming and growing inside the positive-mass black hole. Of course, contributions of several disconnected parts may be present. Nonetheless, we expect our simplified description to give the leading contribution.

One nice feature of our model is that one of the main assumptions of the Central Dogma—the existence of a description of the black hole in terms of $N∼S_{BH}$ quantum states—certainly applies. Although the precise mechanism that allows information to escape from the black hole interior is not fully clear (this would require a precise description of AdS${}_{2}$ quantum gravity), the existence of an underlying dual CFT dynamics drastically improves our understanding of the process of the information flow. On the one hand, our outcome represents an independent confirmation of several interesting results which recently appeared in the literature [37,51,54], in which the Page curve for 2D black holes is derived either using higher-dimensional theories or an holographic entropy formula. On the other hand, our model allows us to compute the Page curve for the EE entropy in a closed form and in a rather simple manner, and to keep track of the quantum mechanical correlations between the interior and the exterior of the black hole in a natural way, by working entirely in the 2D theory context.

Another interesting feature of our model and our results is that they explain in a simple and intuitive way how geometry and quantum entanglement are both essential to save unitarity in the black hole evaporation process. This happens in a way which is fully consistent with the ER = EPR proposal and the emergence of the classical space-time structure out of quantum entanglement [50]. The informational content of the eternal JT black holes can be considered as the information contained in one of the two edges of the maximally extended AdS${}_{2}$ space-time [69]. As evaporation proceeds, after the Page time, information extraction from the hole occurs in two steps. During the first one, which terminates with the black hole setting down to the AdS${}_{0}$ vacuum, only quantum correlations in one edge of AdS${}_{2}$ are reconstructed. In the second one, characterized by the phase transition from AdS${}_{0}$ to the CDV (the two-edged AdS${}_{2}$), the full quantum correlations in the two edges are restored, leaving behind a final pure state for the radiation and a regular space-time geometry. Unfortunately, our effective theory cannot be used to describe this second step, in particular to explain how quantum correlations may emerge between classically disconnected regions of space-time. It is quite obvious that, for these purposes, a full AdS${}_{2}$ quantum gravity theory is needed. Actually, the phase transition by itself is most likely a signal of the breakdown of our effective description and of the reorganization of the relevant DOF.

Apart from its simplicity, our model has also other drawbacks. It is a 2D model with AdS asymptotics and it is not clear to what extent it may capture features of asymptotically flat 4D black holes. The JT model appears in a variety of cases as a description of the near-extremal near-horizon regime of charged and rotating 3D, 4D (and also D-dimensional) black holes (see, e.g., [42,43,62,83,84,85,86,87,88]). Thus, our model is a good approximation for this kind of black holes in the aforementioned regime, but its validity for generic black holes has to be further investigated.

## Data Availability

Not applicable.

## References

[1] Hawking S.W. (1975). Particle Creation by Black Holes. Commun. Math. Phys..

[2] Hawking S.W. (1974). Black hole explosions. Nature.

[3] Hawking S.W. (1976). Breakdown of Predictability in Gravitational Collapse. Phys. Rev. D.

[4] Polchinski J. (2016). The Black Hole Information Problem. arXiv.

[5] Harlow D. (2016). Jerusalem Lectures on Black Holes and Quantum Information. Rev. Mod. Phys..

[6] Mathur S.D. (2009). The Information paradox: A Pedagogical introduction. Class. Quant. Grav..

[7] Unruh W.G., Wald R.M. (1995). On evolution laws taking pure states to mixed states in quantum field theory. Phys. Rev. D.

[8] Unruh W.G., Wald R.M. (2017). Information Loss. Rept. Prog. Phys..

[9] Banks T., Susskind L., Peskin M.E. (1984). Difficulties for the Evolution of Pure States Into Mixed States. Nucl. Phys. B.

[10] Chen P., Ong Y.C., Yeom D.H. (2015). Black Hole Remnants and the Information Loss Paradox. Phys. Rept..

[11] Mathur S.D. (2005). The Fuzzball proposal for black holes: An Elementary review. Fortsch. Phys..

[12] Susskind L. (1995). The World as a hologram. J. Math. Phys..

[13] Maldacena J.M. (1999). The Large N limit of superconformal field theories and supergravity. Int. J. Theor. Phys..

[14] Gubser S.S., Klebanov I.R., Polyakov A.M. (1998). Gauge theory correlators from noncritical string theory. Phys. Lett. B.

[15] Witten E. (1998). Anti-de Sitter space and holography. Adv. Theor. Math. Phys..

[16] Aharony O., Gubser S.S., Maldacena J.M., Ooguri H., Oz Y. (2000). Large N field theories, string theory and gravity. Phys. Rept..

[17] Almheiri A., Marolf D., Polchinski J., Sully J. (2013). Black Holes: Complementarity or Firewalls?. J. Energy Phys..

[18] Almheiri A., Marolf D., Polchinski J., Stanford D., Sully J. (2013). An Apologia for Firewalls. J. Energy Phys..

[19] Witten E. (1998). Anti-de Sitter space, thermal phase transition, and confinement in gauge theories. Adv. Theor. Math. Phys..

[20] Strominger A. (1998). Black hole entropy from near horizon microstates. J. Energy Phys..

[21] Cadoni M., Mignemi S. (1999). Entropy of 2-D black holes from counting microstates. Phys. Rev. D.

[22] Almheiri A., Hartman T., Maldacena J., Shaghoulian E., Tajdini A. (2020). The entropy of Hawking radiation. arXiv.

[23] Giddings S.B. (2012). Models for unitary black hole disintegration. Phys. Rev. D.

[24] Giddings S.B., Shi Y. (2013). Quantum information transfer and models for black hole mechanics. Phys. Rev. D.

[25] Giddings S.B. (2013). Nonviolent nonlocality. Phys. Rev. D.

[26] Giddings S.B. (2021). A ”black hole theorem”, and its implications. arXiv.

[27] Zhang B., Cai Q.Y., Zhan M.S., You L. (2013). Information conservation is fundamental: Recovering the lost information in Hawking radiation. Int. J. Mod. Phys. D.

[28] Corda C. (2015). Time dependent Schrödinger equation for black hole evaporation: No information loss. Ann. Phys..

[29] Page D.N. (1993). Information in black hole radiation. Phys. Rev. Lett..

[30] Page D.N. (2013). Time Dependence of Hawking Radiation Entropy. JCAP.

[31] Penington G., Shenker S.H., Stanford D., Yang Z. (2019). Replica wormholes and the black hole interior. arXiv.

[32] Almheiri A., Hartman T., Maldacena J., Shaghoulian E., Tajdini A. (2020). Replica Wormholes and the Entropy of Hawking Radiation. J. Energy Phys..

[33] Ryu S., Takayanagi T. (2006). Holographic derivation of entanglement entropy from AdS/CFT. Phys. Rev. Lett..

[34] Engelhardt N., Wall A.C. (2015). Quantum Extremal Surfaces: Holographic Entanglement Entropy beyond the Classical Regime. J. Energy Phys..

[35] Penington G. (2020). Entanglement Wedge Reconstruction and the Information Paradox. J. Energy Phys..

[36] Almheiri A., Engelhardt N., Marolf D., Maxfield H. (2019). The entropy of bulk quantum fields and the entanglement wedge of an evaporating black hole. J. Energy Phys..

[37] Almheiri A., Mahajan R., Maldacena J., Zhao Y. (2020). The Page curve of Hawking radiation from semiclassical geometry. J. Energy Phys..

[38] Almheiri A., Mahajan R., Maldacena J. (2019). Islands outside the horizon. arXiv.

[39] Jackiw R. (1985). Lower Dimensional Gravity. Nucl. Phys. B.

[40] Teitelboim C. (1983). Gravitation and Hamiltonian Structure in Two Space-Time Dimensions. Phys. Lett. B.

[41] Grumiller D., Kummer W., Vassilevich D.V. (2002). Dilaton gravity in two-dimensions. Phys. Rept..

[42] Cadoni M., Mignemi S. (1994). Classical and semiclassical properties of extremal black holes with dilaton and modulus fields. Nucl. Phys. B.

[43] Cadoni M., Mignemi S. (1995). Nonsingular four-dimensional black holes and the Jackiw-Teitelboim theory. Phys. Rev. D.

[44] Cadoni M., Ciulu M., Tuveri M. (2018). Symmetries, Holography and Quantum Phase Transition in Two-dimensional Dilaton AdS Gravity. Phys. Rev. D.

[45] Cadoni M. (2007). Entanglement entropy of two-dimensional Anti-de Sitter black holes. Phys. Lett. B.

[46] Hubeny V.E., Rangamani M., Takayanagi T. (2007). A Covariant holographic entanglement entropy proposal. J. Energy Phys..

[47] Giataganas D., Tetradis N. (2019). Entanglement entropy, horizons and holography. Phys. Lett. B.

[48] Van Raamsdonk M. (2010). Building up spacetime with quantum entanglement. Gen. Rel. Grav..

[49] Maldacena J., Susskind L. (2013). Cool horizons for entangled black holes. Fortsch. Phys..

[50] Van Raamsdonk M. (2009). Comments on quantum gravity and entanglement. arXiv.

[51] Gautason F.F., Schneiderbauer L., Sybesma W., Thorlacius L. (2020). Page Curve for an Evaporating Black Hole. J. Energy Phys..

[52] Callan C.G., Giddings S.B., Harvey J.A., Strominger A. (1992). Evanescent black holes. Phys. Rev. D.

[53] Russo J.G., Susskind L., Thorlacius L. (1992). The Endpoint of Hawking radiation. Phys. Rev. D.

[54] Verheijden E., Verlinde E. (2021). From the BTZ black hole to JT gravity: Geometrizing the island. arXiv.

[55] Goto K., Hartman T., Tajdini A. (2020). Replica wormholes for an evaporating 2D black hole. arXiv.

[56] Marolf D., Maxfield H. (2020). Observations of Hawking radiation: The Page curve and baby universes. arXiv.

[57] Kim W., Nam M. (2020). Entanglement entropy of asymptotically flat non-extremal and extremal black holes with an island. arXiv.

[58] Hollowood T.J., Kumar S.P. (2020). Islands and Page Curves for Evaporating Black Holes in JT Gravity. J. Energy Phys..

[59] Anegawa T., Iizuka N. (2020). Notes on islands in asymptotically flat 2d dilaton black holes. J. Energy Phys..

[60] Bousso R., Shahbazi-Moghaddam A. (2021). Island Finder and Entropy Bound. Phys. Rev. D.

[61] Almheiri A., Polchinski J. (2015). Models of AdS_2_ backreaction and holography. J. Energy Phys..

[62] Achucarro A., Ortiz M.E. (1993). Relating black holes in two-dimensions and three-dimensions. Phys. Rev. D.

[63] Maldacena J.M., Michelson J., Strominger A. (1999). Anti-de Sitter fragmentation. J. Energy Phys..

[64] Christensen S.M., Fulling S.A. (1977). Trace Anomalies and the Hawking Effect. Phys. Rev. D.

[65] Unruh W.G. (1976). Notes on black hole evaporation. Phys. Rev. D.

[66] Hawking S.W., Ellis G.F.R. (2011). The Large Scale Structure of Space-Time.

[67] Cadoni M., Cavaglia M. (2001). Open strings, 2-D gravity and AdS / CFT correspondence. Phys. Rev. D.

[68] Cadoni M., Cavaglia M. (2001). Two-dimensional black holes as open strings: A New realization of the AdS / CFT duality. Phys. Lett. B.

[69] Maldacena J.M. (2003). Eternal black holes in anti-de Sitter. J. Energy Phys..

[70] Birrell N.D., Davies P.C.W. (1984). Quantum Fields in Curved Space.

[71] Horowitz G.T., Maldacena J.M. (2004). The Black hole final state. J. Energy Phys..

[72] Papadodimas K., Raju S. (2013). An Infalling Observer in AdS/CFT. J. Energy Phys..

[73] Avery S.G., Chowdhury B.D., Puhm A. (2013). Unitarity and fuzzball complementarity: ‘Alice fuzzes but may not even know it!’. J. Energy Phys..

[74] Verlinde E., Verlinde H. (2013). Passing through the Firewall. arXiv.

[75] ‘t Hooft G. (2016). Black hole unitarity and antipodal entanglement. Found. Phys..

[76] Liu H., Vardhan S. (2021). A dynamical mechanism for the Page curve from quantum chaos. J. Energy Phys..

[77] Fiola T.M., Preskill J., Strominger A., Trivedi S.P. (1994). Black hole thermodynamics and information loss in two-dimensions. Phys. Rev. D.

[78] Cadoni M., Tuveri M., Sanna A.P. (2020). Long-Range Quantum Gravity. Symmetry.

[79] Tuveri M., Cadoni M. (2019). Galactic dynamics and long-range quantum gravity. Phys. Rev. D.

[80] Susskind L., Witten E. (1998). The Holographic bound in anti-de Sitter space. arXiv.

[81] Alonso-Serrano A., Visser M. (2018). Entropy/information flux in Hawking radiation. Phys. Lett. B.

[82] Mück W. (2016). Hawking radiation is corpuscular. Eur. Phys. J. C.

[83] Giddings S.B., Strominger A. (1992). Dynamics of extremal black holes. Phys. Rev. D.

[84] Trivedi S.P. (1993). Semiclassical extremal black holes. Phys. Rev. D.

[85] Almheiri A., Kang B. (2016). Conformal Symmetry Breaking and Thermodynamics of Near-Extremal Black Holes. J. Energy Phys..

[86] Nayak P., Shukla A., Soni R.M., Trivedi S.P., Vishal V. (2018). On the Dynamics of Near-Extremal Black Holes. J. Energy Phys..

[87] Moitra U., Trivedi S.P., Vishal V. (2019). Extremal and near-extremal black holes and near-CFT_1_. J. Energy Phys..

[88] Moitra U., Sake S.K., Trivedi S.P., Vishal V. (2019). Jackiw-Teitelboim Gravity and Rotating Black Holes. J. Energy Phys..

